# Interpersonal Violence and Psychotic-Like Experiences: The Mediation of Ideas of Reference, Childhood Memories, and Dissociation

**DOI:** 10.3390/ijerph17124587

**Published:** 2020-06-25

**Authors:** Sandra Fernández-León, Juan F. Rodríguez-Testal, María L. Gutiérrez-López, Cristina Senín-Calderón

**Affiliations:** 1 Clinical Mental Health Management Unit, Hospital Juan Ramón Jiménez, 21005 Huelva, Spain; leonhiss@gmail.com; 2 Personality, Evaluation and Psychological Treatment Department, University of Seville, 41018 Seville, Spain; 3 La Palma del Condado Mental Health Unit, Infanta Elena Hospital, 21700 Huelva, Spain; marisagulo@hotmail.com; 4 Department of Psychology, University of Cadiz, Cadiz 11071, Spain; cristina.senin@uca.es

**Keywords:** psychosis, interpersonal violence, ideas of reference, aberrant salience, dissociation, childhood memories

## Abstract

Previous studies have demonstrated the relationship between the accumulation of situations involving interpersonal violence (IV) and psychotic-like experiences. This study explored whether IV is related to aberrant salience (AS), using a sequential mediation model that included memories of relationship with parents (submission, devaluation, and threat; Early Life Experiences Scale (ELES)), ideas of reference (IR), and dissociative symptoms (absorption and depersonalization), and whether the patient/nonpatient condition moderated this effect. The sample was made of 401 participants (including 43 patients with psychotic disorders) aged 18 to 71 years (*M*age = 30.43; *SD* = 11.19). Analysis of a serial multiple mediator model revealed that IR, ELES, absorption, and depersonalization fully mediated the effect of IV on AS, explaining 39% of the variance, regardless of the patient/nonpatient condition. The indirect paths, which place IR and dissociation (especially absorption, the variable to which the IR and ELES lead) in a primordial position for being related to AS, are discussed. This continuum model could be useful for understanding processes related to the onset of psychosis unmoderated by the patient/nonpatient condition.

## 1. Introduction

There is a certain consensus on the relationship between childhood trauma and the onset of psychosis [1]. Situations involving violence, such as physical or sexual abuse, were related to positive symptoms, particularly hallucinations or persecutory ideation [2,3,4]. However, violence often does not appear as a single type [5], nor at a single moment in time. That is, it might be present during childhood, maintained, accumulated, or begin later, during adolescence, or adulthood [6]. It is assumed that complex relationships exist between childhood traumatic experiences, derived psychotic experiences, and victimization in childhood or adulthood [7]. Thus, it might be relevant to consider interpersonal violence globally (physical or sexual abuse, bullying, aggression, and so on) [8], rather than separately, as violence is commonly expressed in several ways on the same person.

What might be essential to these situations is that it affects interpersonal contact. The context in which such violence arises, often in the family, emotionally sensitizes the person and reinforces anticipation when faced with threat or harm [9,10]. The continuity and accumulation of events involving violence might intensify psychotic-like experiences (PLE) or psychotic symptoms, if already present [11]. They also activate memories of such situations and responses of submission, devaluation or defense from threat [12]. One of the relationships well-established in research is between traumatic events and dissociation. Dissociation might be the response of a maladjusted child, youth, or adult, to their resources being overwhelmed [13]. Recent reviews agree on the relationship between dissociation (e.g., absorption and depersonalization) and positive symptoms, such as hallucination, paranoia, or delusions [14]. This relationship was verified by analyzing PLE with both patients and nonclinical populations [15]. The mediating role of dissociation in the trauma/psychosis relationship therefore seems clear.

Other proposed mediating processes between traumatic events (related to violence) and psychotic symptoms have to do with beliefs about the self and cognitive functioning in the social world [16]. In fact, a major position was given to alterations of the self in understanding the development of schizophrenia in clinical samples, participants in high-risk mental states, and the general population [17,18,19].

In this sense, another relevant process is ideas of reference (IR), one of the components related to beliefs about the self [20], which might be the basis for developing later delusional ideation [21]. IR is often dealt with as PLE, however, it is likely that their importance depends on their persistence and the distress they cause before crystalizing in delusion [22]. It would then make sense to confirm a relationship between general interpersonal violence and the emergence of IR [23], along with dissociative responses to the capacity for adjustment to the threatening meaning of experiences being overwhelmed.

Summarizing, and taking the presence of interpersonal violence as a starting point, this study poses the possibility that this set of situations favors the appearance of a more automatic defensive cognitive process such as IR, as an expression of self-disturbances [19,24]. At the same time, such violence could activate memories of the relationship with one’s parents, causing submission, devaluation, or defense for coping with the threat [12]. These mediator variables would also occur sequentially with other mediator variables, such as absorption and depersonalization, as confirmed in analyses with these dissociative variables [15,25]. In this set of mediator variables, especially due to the proximity of dissociation, aberrant salience as a PLE is proposed as a triggering relationship. This measure would represent the global and diffuse changes prior to the development of persistent psychotic experiences and delusional development, and therefore, would be analyzable in both general and clinical populations [26,27].

**Figure 1:**
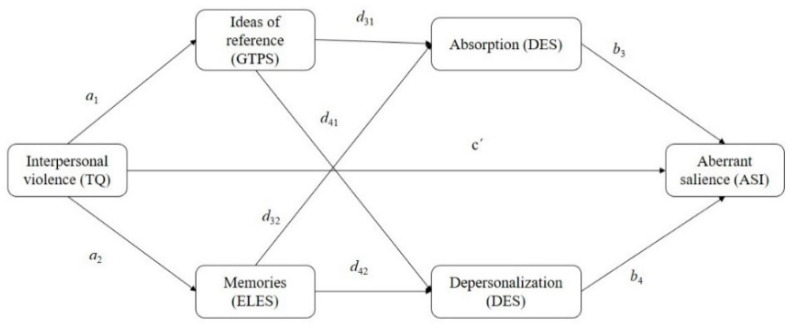
Path diagram of hypotheses 1 and 2; the conceptual model. Note: a_1_ = path from Interpersonal Violence to Ideas of Reference; a_2_ = path from Interpersonal Violence to Memories; d_31_ = path from Ideas of Reference to Absorption; d_41_ = Path from Ideas of Reference to Depersonalization; d_32_ = path from Memories to Absorption; d_42_ = Path from Memories to Depersonalization; b_3_ = path from Absorption to Aberrant Salience; b_4_ = path from Depersonalization to Aberrant Salience. C′ = direct effect of X on Y.

From the perspective of a psychosis continuum, with a sample of the general population and a sample of patients with schizophrenia spectrum disorders and bipolar disorders evaluated in a hospital unit, a novel sequence was proposed as an approach to knowledge about the onset of psychosis. A statistically significant positive relationship between interpersonal violence and the emergence of aberrant salience (ASI) was predicted (hypothesis 1). This total effect would be mediated by the relationship of interpersonal violence and the presence of IRs (GPTS), as well as the memory of the relationship with one’s parents (submission, devaluation, and threat; Early Life Experiences Scale (ELES)). Triggering of IRs and childhood memories (ELES) would be positively and statistically significantly related to the appearance of absorption and depersonalization (DES). These mediator variables would then be statistically significantly related to the appearance of aberrant salience (ASI) (hypothesis 2). This relationship of the mediator variables counts on the patient/nonpatient condition as a variable moderating the relationship between interpersonal violence and aberrant salience (ASI) (hypothesis 3). Hypotheses 1 and 2 are shown in Figure 1.

## 2. Material and Methods

### 2.1. Participants

The sample comprised 401 participants (61.80% women) aged 18 to 71 (*M*age = 30.43; *SD* = 11.20). Of these, 43 (48.8% women; *M*age = 34.81, *SD* = 13.03) were patients in the Inpatient Mental Health Unit at the Juan Ramón Jiménez Hospital (Huelva, Spain). Patient diagnoses by the DSM-5 criteria [28] were—schizophrenia (*n* = 27), delusional disorder (*n* = 5), and bipolar disorders (*n* = 11). The rest of the participants were recruited from the general population through snowball sampling (56.6% women, *M*age = 29.90, *SD* = 10.85). There was no statistically significant difference in sex between samples (*χ*2(1, 401)= 3.45, *p* = 0.063), although there was in age (*t*(399) = 2.74, *p* = 0.022), therefore, age was taken as a covariant in the statistical analysis.

### 2.2. Measures

Trauma was measured by the Trauma Questionnaire (TQ) [29]; Spanish validation carried out by Bobes et al. [30]. In 18 “yes”/”no” items, it evaluated the number and type of traumas undergone during one’s life, the age at which they occurred, and duration. This study employed the sum of violent traumatic events undergone—abuse, incest, rape, threat, aggression, combat, hostage, and kidnapping. The Cronbach’s alpha for the complete scale in the Spanish validation was 0.99. In the sample in this study, the Cronbach’s alpha was 0.51.

Paranoia was measured by the Green et al. Paranoid Thought Scales (GPTS) [31]. This was a 32-item scale for self-informed evaluation of persecutory ideas in general and clinical populations. The answer format ranged from “1” (Not at all) to “5” (Totally). It had two subscales that evaluated the ideas of social reference and persecution. In this study, the subscale for ideas of reference was employed. In the Spanish validation, the Cronbach’s alpha was 0.92 for this factor [32]. With the sample in this study, the Cronbach’s α was also 0.92.

The Early Life Experiences Scale (ELES) [33], adapted to Spanish by León-Palacios et al. [34], was used to evaluate the memory of threats and subordination experienced in relationships with parents during childhood. It had 15 items scored on a Likert-type scale from “1” (completely false) to “5” (completely true). It contained three factors that referred to feelings of threat, submission, and devaluation. In the Spanish validation, internal consistency was adequate on all three factors, *α* = 0.90 (threat), *α* = 0.86 (s0ubmission), and *α* = 0.81 (devaluation). In this study, the total score was employed, with the study sample *α* = 0.83.

The Aberrant Salience Inventory (ASI) [35], adapted to Spanish by Fernández-León et al. [36], was used as a measure of proneness to psychosis, which evaluated the assignment of significance or importance to normally irrelevant internal and external stimuli. It had 29 items with a “true”/”false” answer choice. It consisted of five factors, but the total scale score could be used as recommended by its author. The Spanish validation had adequate internal consistency (ordinal alpha = 0.95). For the sample in this study, the Cronbach’s alpha was 0.90.

Dissociation was measured by the Dissociative Experiences Scale II (DES-II) [37]; validated in Spanish by Icarán et al. [38]. This was a 28-item self-report scale evaluating the frequency of dissociative experiences. It contained three factors—absorption, derealization/depersonalization, and amnesia. The answer format was evaluated in percentages from 0% (never) to 100% (very often). In this study, the absorption and derealization/depersonalization factors were used. The authors found a Cronbach’s α of 0.78 and 0.65, respectively. With the study sample, the *α* was 0.76 and 0.68, respectively.

### 2.3. Procedure

Tests were administered to the participants in the general population group in an online format (without missing data). The patients filled out the tests on paper during their hospitalization. All participants gave their written informed consent. A clinical case was excluded due to incomplete data. The study was approved by the Andalusia Regional Government Ethics Committee (Spain) (PI010/16).

### 2.4. Data Analysis

The descriptive statistics and Pearson’s correlations of the study variables were found. The working hypothesis was tested by serial mediation analysis with the PROCESS macro (Model 6 [39]), which evaluated the indirect effect of the number of situations of interpersonal violence experienced (IV) on aberrant salience (AS), through ideas of reference (IR) and absorption (i.e., the a1–d31–b3 path; Figure 1), through IR and depersonalization (i.e., the a1–d41–b4 path; Figure 1), through childhood memories (submission, threat, and devaluation) (ELES) and absorption (i.e., the a2–d32–b3 path; Figure 1), and through ELES and depersonalization (i.e., the a2–d42–b4 path; Figure 1). Then a mediation analysis with moderation was performed (Model 5 [39]), where the group variable was included (patients vs. nonpatient) as a moderator of the direct effect of the IV variable on AS. In the analyses of Models 6 and 5, age was included as a covariant. We used 5000 bootstrapping resamples to produce 95% confidence intervals for the indirect effect. If the confidence interval did not contain zero, one could conclude that mediation was significant.

## 3. Results

### 3.1. Preliminary Analysis

Table 1 shows the descriptive statistics and Pearson’s correlations of the study variables. Statistically significant correlations with medium to high effect size were found between interpersonal violence and childhood memories (ELES), as well as between ideas of reference (IR) and dissociative symptoms (absorption and depersonalization), in both samples. The correlations of aberrant salience (AS) with dissociative symptoms and IR were statistically significant, with a medium to large effect size in the general population group and a moderate one in the group of patients. Statistically significant correlations were found between absorption and age, but only with a small effect size in the general population group.

### 3.2. Serial Mediation Analyses

Interpersonal violence (IV) was positively associated with aberrant salience (AS), explaining 6% of the variance, and supporting hypothesis 1(H1) (total effect: β = 1.49, SE = 0.35, 95% CI [0.81, 2.17]). As observed in Table 2, there were three significantly indirect paths from IV to AS, through IR and absorption, through IR and depersonalization, and through ELES and absorption. The path analyzing the indirect effect of IV on AS through ELES and depersonalization was not statistically significant. When the mediating variables were controlled for, the direct effect of IV on AS was not statistically significant, which showed full mediation. These results suggest serial multiple mediation, explaining 39% of the variance and partially confirming hyphothesis 2 (H2). The age covariant was statistically significant.

### 3.3. Test of Mediation with a Moderated Direct Effect

A moderated mediation analysis was carried out to find out whether the relationship between IV on AS could be moderated by the patient vs. nonpatient condition. The results did not support our third hypothesis, showing that having experienced interpersonal violence was associated with a higher score on AS, regardless of group (Conditional direct effect—Nonpatients: β = 0.62, SE = 0.33, 95% IC [−0.02, 1.27]; and Patients: β = −1.07, SE = 0.63, 95% CI [−2.30, 0.15]). The overall model accounted for 43% of the variance in the ASI total score, indicating a large effect size (Cohen’s f2 = 0.75 [40]).

## 4. Discussion

Psychosis is one of the most suffering-inducing clinical conditions for the people who experience it and the setting where it takes place. Knowing the different processes involved and their relationship is crucial for the detection, approach, and prevention of psychosis.

This study analyzed situations involving interpersonal violence and the age at which these situations occurred, to verify their relationship with aberrant salience (ASI). Aberrant salience, as a measure, refers to the significant changes in an experiential state of strange experiences, without any specific meaning [26], such as psychotic-like experiences (PLE). Although certain situations involving interpersonal violence are often related to specific positive symptoms, a set of violent situations was considered here because they change the way in which trust between persons are integrated [17,41]. The proposal for onset of psychosis, understood as a process, not necessarily the first episode, was approached by emphasizing the perception of the world as hostile and threatening, affecting not only children and adolescents, but also adults [6].

Consideration of the relationship of situations involving different forms of interpersonal violence and psychosis is supported in the literature [16]. Thus, as a total effect, the first hypothesis was met. The emotional impact or psychobiological repercussions associated with the type of situation experienced means changes in the person and in his/her relations with others, probably favoring a state of continual stress. Evaluation of relations with others, sometimes, the same people who have led to these situations, and adoption of coping strategies to handle them, must exert an important role, as corroborated by the relationship observed between the set of situations and childhood memories (ELES) [12].

In this context, it makes sense for ideas of reference (IRs) to emerge as a form of social cognition [21], due to their relationship with situations perceived as threatening. As IRs take place in normal functioning, in populations with at-risk mental states and in clearly pathological functioning [21], they fit in with a time when interpersonal susceptibility is triggered, although it is still a very unspecific and little-developed process [42]. The permanence of this state of alarm, particularly the persistence of IRs, is what is usually related to psychotic processes [43]. Although this design cannot ensure continuity in the presence of IRs, it is nevertheless true that the relationship with situations involving violence is solid, and it is not disregarded that such situations are repeated or that they maintain their effect. IRs and memories of the relationship with one’s parents (ELES) are related sequentially with the dissociative response (absorption and depersonalization), more clearly in the case of IRs, as observed previously [25]. Both forms of dissociation were significantly related to aberrant salience (PLE), and therefore, this confirmed hypothesis 2.

This sequence of mediator variables showed that IRs might have an outstanding role in what might later become a delusional development, consolidated with the emergence of aberrant salience. Thus, the two constructs are close, but refer to different cognitive processes [36]. IRs probably represent self-disturbance [22], proposed as being essential to characterizing psychosis in general and schizophrenia in particular [19]. It is therefore possible that IRs first appear as a reaction, a defensive element more typical of social cognition, and it later becomes more disorganized, as with the participation of dissociation, the stimulating salience increases [44,45]. In other studies, aberrant salience was considered to be a mediator variable [24,46], but it should not be discarded that these variables provide feedback to each other, or that they represent two moments with different degrees of disorganization (dissociation).

Some authors have emphasized that one of the key elements in the relationship between trauma and psychosis is emotion, particularly anxiety [47]. Participation of emotional variables in the genesis of psychosis is a consolidated premise [48], although it was not tested in this study. In this study, a major role in the sequence of mediator variables was given to the known relationship between dissociation (absorption and depersonalization) and the psychotic process [14]. Other authors, however, believe it does not refer as much to dissociation or depersonalization as to the destructuring of the more global personality [49]. Others have found that dissociation intervenes in the relationship of traumatic events with psychosis, but in parallel, and is essential in cases of high cumulative trauma, due to its relationship with aberrant salience [50].

Memories of the relationship with parents (ELES) [12] were also included in this set, although their later relationship was mainly with absorption, and significantly, with IRs. Some studies following up on samples of patients have attributed less importance to the memories of such events, and more to the mediating role of activating, compared to dissociative, states [23].

The role of the group condition (with/without psychosis) was also analyzed to verify whether it had a moderating role. However, hypothesis 3 was discarded, rejecting the idea of moderation, although in both cases, some probability was observed. Perhaps this sequence occurred throughout the continuum, without the clinical condition being clearly differentiating in this sense.

## 5. Conclusions

The results of this study must be interpreted with caution, keeping its limitations in mind. In the first place, the cross-sectional design limits the generalization of the results. It was not a proposal that involves causality, although it might suggest a relationship of variables to be considered in the study of psychosis. It was based on the concept of a continuum, where the people without pathology can show the established relationships to a lesser extent than those people who are at risk or have a diagnosis of a psychotic disorder. The use of self-informed instruments was also a limitation, because they could lead to a bias in understanding the questions or in the evocation of traumatic events. However, in other studies, clinical assessments of PLEs were shown to be rather precise, even when evaluated at different times [51]. With respect to traumatic situations, it might be a limitation to consider these as a whole, due to the lack of specificity and low reliability. Other studies have shown that it is difficult to really establish pure forms of trauma [52], and more usually these are combined, or as mentioned above, understanding that the impact they have is due to the evaluation made by the person who suffers from them has a greater bearing on it. Another limitation refers to the participation of a relatively small group of patients for testing the role of the group variable as a moderator of the original relationship. This condition should be explored with a larger sample, although the fact that it is a group of participants evaluated in a hospital unit, provides value to the consideration of psychosis on a continuum right at the time of emergence or reappearance of the psychotic process. Although this design did not take into consideration the effect of treatments, the clinical characteristics, or other indicators of these participants, it was a group of choice because there was practically no substance use, very few episodes, and the disorder developed in a very short time.

## Figures and Tables

**Table 1:** Means, standard deviations, and correlation for all variables included in the model.

Variable	1	2	3	4	5	6	7
1	-	0.257	0.412 **	0.215	0.061	−0.008	0.010
2	0.260 **	-	0.235	0.487 **	0.594 **	0.392 **	−0.139
3	0.298 **	0.247 **	-	0.074	0.041	0.107	0.072
4	0.189 **	0.470 **	0.282 **	-	0.793 **	0.337 *	−0.128
5	0.146 **	0.412 **	0.162 **	0.530 **	-	0.307 *	−0.175
6	0.204 **	0.499 **	0.116 **	0.571 **	0.455 **	-	0.062
7	−0.031	−0.009	−0.003	−0.205 **	−0.035	0.075	-
M patients (*SD*) *n* = 43	1.23 (1.39)	38.56 (18.96)	39.81 (13.42)	1.99 (2.68)	1.06 (1.79)	17.74 (7.19)	34.81 (13.03)
M general population (*SD*), *n* = 358	0.53 (0.89)	26.79 (11.28)	35.77 (10.28)	2.49 (1.69)	0.37 (.83)	10.77 (6.68)	27.16 (10.22)

1. TQ = Interpersonal Violence, 2. GTPS = Ideas of reference, 3. ELES = Total, 4. DES = Absorption, 5. DES = Depersonalization, 6. ASI = Aberrant salience, and 7. Age. The correlations for the group of patients are given in the upper diagonal and in the lower diagonal those corresponding to the general population. M = Mean; *SD* = Standard Deviation; ** *p* < 0.01. * < *p* 0.05.

**Table 2:** Total, direct, and indirect effect.

Criterion: Interpersonal Violence	Coeff.	SE	t	LLCI	ULCI
Total effect of X on Y	1.49	0.35	4.29 **	0.81	2.17
Total direct effect of X on Y	0.45	0.30	1.46	−0.15	1.05
Age (covariate)	0.07	0.03	2.19 *	0.01	0.13
Indirect effect(s) of IV on AS	Coeff.	Boot SE		BootLLCI	BootULCI
Total indirect effects of IV on AS	1.04 **	0.28	-	0.50	1.60
Indirect effect 1: X → M1→ M3 → Y	0.10 **	0.05	-	0.02	0.21
Indirect effect 2: X → M1→ M4 → Y	0.01	0.01	-	−0.04	0.02
Indirect effect 3: X → M2→ M3 → Y	0.21 **	0.08	-	0.09	0.39
Indirect effect 4: X → M2→ M4 → Y	0.05 **	0.03	-	0.01	0.12

Note: X = Interpersonal Violence; M1 = Childhood Memories (ELES); M2 = Ideas of Reference; M3 = Absorption; M4 = Depersonalization, and Y = Aberrant Salience. **p < 0.01 *p < 0.05. Coeff. = Beta Coefficient; SE= Standard Error; t = Student´s *t*-test; LLCI= Lower Level of Confidence Interval; ULCI = Upper Level of Confidence Interval.
